# Direct interaction of beta-amyloid with Na,K-ATPase as a putative regulator of the enzyme function

**DOI:** 10.1038/srep27738

**Published:** 2016-06-14

**Authors:** Irina Yu. Petrushanko, Vladimir A. Mitkevich, Anastasia A. Anashkina, Alexei A. Adzhubei, Ksenia M. Burnysheva, Valentina A. Lakunina, Yulia V. Kamanina, Elena A. Dergousova, Olga D. Lopina, Omolara O. Ogunshola, Anna Yu. Bogdanova, Alexander A. Makarov

**Affiliations:** 1 Engelhardt Institute of Molecular Biology, Russian Academy of Sciences, Vavilov St. 32, 119991 Moscow, Russia; 2 Faculty of Biology, M.V. Lomonosov Moscow State University, 119234 Moscow, Russia; 3 Institute of Veterinary Physiology, Vetsuisse Faculty, and the Zurich Center for Integrative Human Physiology (ZIHP), University of Zurich, CH-8057 Zurich, Switzerland

## Abstract

By maintaining the Na^+^ and K^+^ transmembrane gradient mammalian Na,K-ATPase acts as a key regulator of neuronal electrotonic properties. Na,K-ATPase has an important role in synaptic transmission and memory formation. Accumulation of beta-amyloid (Aβ) at the early stages of Alzheimer’s disease is accompanied by reduction of Na,K-ATPase functional activity. The molecular mechanism behind this phenomenon is not known. Here we show that the monomeric Aβ(1-42) forms a tight (*K*_*d*_ of 3 μM), enthalpy-driven equimolar complex with α1β1 Na,K-ATPase. The complex formation results in dose-dependent inhibition of the enzyme hydrolytic activity. The binding site of Aβ(1-42) is localized in the “gap” between the alpha- and beta-subunits of Na,K-ATPase, disrupting the enzyme functionality by preventing the subunits from shifting towards each other. Interaction of Na,K-ATPase with exogenous Aβ(1-42) leads to a pronounced decrease of the enzyme transport and hydrolytic activity and Src-kinase activation in neuroblastoma cells SH-SY5Y. This interaction allows regulation of Na,K-ATPase activity by short-term increase of the Aβ(1-42) level. However prolonged increase of Aβ(1-42) level under pathological conditions could lead to chronical inhibition of Na,K-ATPase and disruption of neuronal function. Taken together, our data suggest the role of beta-amyloid as a novel physiological regulator of Na,K-ATPase.

Na,K-ATPase creates the Na^+^ and K^+^ transmembrane gradient vital for all animal cells, it also is a receptor for cardiotonic steroids, regulating cell proliferation and apoptosis. Na,K-ATPase in neurons consumes up to 80% of ATP, since it not only sustains the Na^+^, K^+^ gradient, but generates the action potential, maintaining the cell electrotonic characteristics^1^. Disruption of ion homeostasis and osmotic balance may hinder the normal electrotonic properties of dendrites by blocking intracellular signaling and contributing to degeneration of neurons^2^. Malfunction of Na,K-ATPase underlies a series of pathologies, such as ischemic tissue damage, cancer and neurodegenerative diseases, such as Alzheimer’s disease (AD). AD is the most widely occurring neurodegenerative disease and is diagnosed in approximately 11% of population older than 65 years and 32% older than 85 years. A therapeutic strategy aimed at increasing the activity of Na,K-ATPase in AD was proposed as symptomatic relief and slowing down the progression of the disease^2^,^3^.

Development of AD is accompanied by the decreased activity of Na,K-ATPase^2^,^3^,^4^,^5^, while the causal link between the two phenomena has not yet been established. In the presence of beta-amyloid (Aβ), a major component of the amyloid plaques formed in AD, the Na,K-ATPase activity in the postmortem brain tissue samples from AD patients is reduced in contrast with the samples from age-matched control^4^; similar correlation was made for the samples from hippocampus and the microsomal fraction of brain tissue of transgenic mice and rats which showed memory deficiencies characteristic of AD^5^. Importantly, reduced activity of Na,K-ATPase was observed only in the areas of the brain where amyloid plaques were formed, i.e. in the hippocampus, but not in the plaques-free cerebellum^2^, suggesting possibility of a direct regulation of the Na,K-ATPase activity by Aβ.

Beta-amyloid (Aβ) is a 36 to 43 amino acids long product of the amyloid precursor protein (APP) hydrolysis^6^, while the 40 and 42 a.a. peptides constitute the main fraction. Aβ in mammals was suggested to be an important factor in the facilitating neuronal growth, cellular survival, the modulation of synaptic function and defense against oxidative stress^7^. The physiological concentrations of Aβ benefit learning and memory processes^8^, however its precise function has not been elucidated until now. Experiments on neuronal cell cultures indicated that at submicromolar concentrations Aβ affects cell differentiation, while at micromolar concentrations Aβ has a cytotoxic effect^9^. Recently it has been shown that amilospheroids, the neurotoxic 10–15-nm spherical Aβ oligomers derived from AD-patients, target neuronal Na,K-ATPase and inhibit its hydrolytic activity^10^. However the amilospheroids are not formed at the early stages of AD, and cannot be the cause of Na,K-ATPase inhibition at these stages of disease. We have hypothesize that the monomeric Aβ can directly interact with Na,K-ATPase and regulate its activity.

In this study using isothermal titration calorimetry we have shown that Aβ(1-42) and Na,K-ATPase form equimolar complex with the dissociation constant (K_d_) 3 μM. We have found that the Na,K-ATPase:Aβ(1-42) complex formation leads to the dose-dependent loss of hydrolytic activity of the enzyme in solution and in neuroblastoma cells SH-SY5Y. Treatment of these cells with Aβ(1-42) also leads to a decrease of Na,K-ATPase transport activity and Src-kinase activation in the first 30 min of incubation. Using fluorescently-labeled Aβ(1-42) we further show that amyloid peptide does not penetrate into cells within this time period. Hence, Aβ(1-42) directly interacts with the extracellular part of Na,K-ATPase and modulates its function. We have built a model of the Aβ(1-42):Na,K-ATPase complex, according to which binding of Aβ(1-42) occurs in the “gap” between the alpha- and beta-subunits, disrupting the function of Na,K-ATPase.

## Results

### Aβ(1-42) interacts with Na,K-ATPase and inhibits its hydrolytic activity in solution

**Figure 1:**
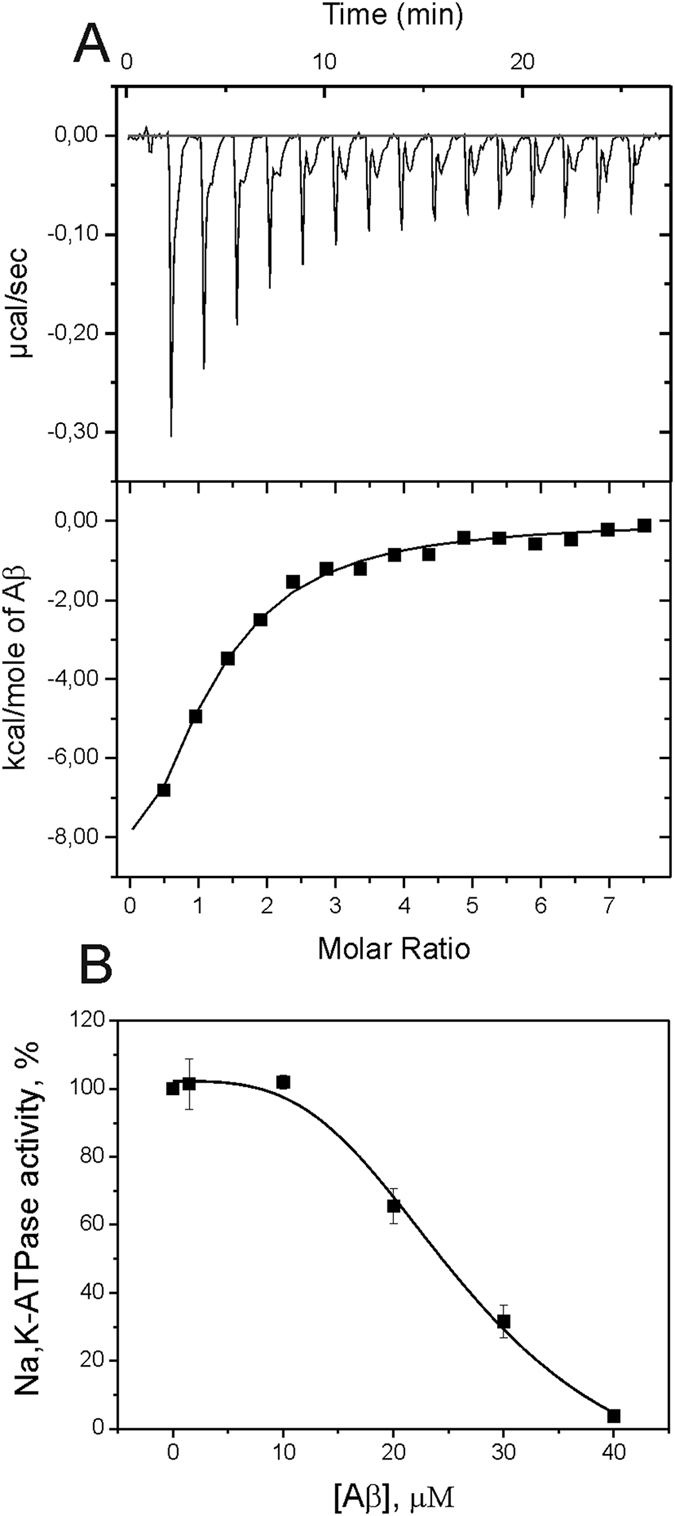
Interaction of Aβ(1-42) with Na,K-ATPase in solution. (**A**) ITC titration curve (upper panel) and binding isotherm (lower panel) for Aβ(1-42) interaction with Na,K-ATPase at 25 °C. (**B**) Dependence of Na,K-ATPase hydrolytic activity on Aβ(1-42) concentration. Enzyme activity without Aβ(1-42) is accepted as 100%.

We used purified preparation of Na,K-ATPase from duck salt glands with high Na,K-ATPase-specific activity which is difficult to achieve when purifying Na,K-ATPase from other tissues^11^. The protein from duck is a homolog of the Na,K-ATPase α1β1 isozyme from human tissues. A typical set of isothermal titration calorimetry (ITC) data for Aβ(1-42) binding to Na,K-ATPase at 25 °C is shown in Fig. 1A. Titration curve was well fitted to a model with one Aβ(1-42) binding site per Na,K-ATPase molecule. Complex formation between Na,K-ATPase and Aβ(1-42) was characterized by the following parameters: dissociation constant (K_d_) 3 ± 1 μM, enthalpy variation (ΔH) −13.2 ± 2.0 kcal/mole and the entropy contribution to binding (TΔS) −5.8 ± 1.2 kcal/mole. These thermodynamic parameters indicate that the binding is enthalpy-driven and is largely caused by electrostatic interactions^12^. The shape of ITC curve allowed to obtain the stoichiometry (N) of Aβ(1-42) binding to Na,K-ATPase equal to 1.1, which demonstrates that Aβ(1-42) is in the monomeric state.

Aβ(1-42) binding to Na,K-ATPase leads to the tremendous reduction of the enzyme hydrolytic activity in the dose-dependent manner (Fig. 1B). At 40 μM Aβ(1-42) almost fully inhibits the ability of Na,K-ATPase to hydrolyze ATP.

The Aβ(1-42) concentration resulting in the enzyme losing half of its hydrolytic activity (IC50) is 24 ± 1 μM. The difference between K_d_ value obtained by ITC and the IC_50_ value can be attributed to the presence of adenine nucleotides in the medium during hydrolytic activity measurements. Similar difference between the K_d_ and IC_50_ values is observed for Na,K-ATPase when it interacts with various cardiotonic steroids^13^. Dilution of the enzyme solution containing 40 μM of Aβ(1-42) down to 10 μM leads to recovery of the Na,K-ATPase activity (Supplementary Figure S1) suggesting that the enzyme inhibition is reversible.

### Aβ(1-42) inhibits hydrolytic and transport activity of Na,K-ATPase in neuroblastoma cells

**Figure 2:**
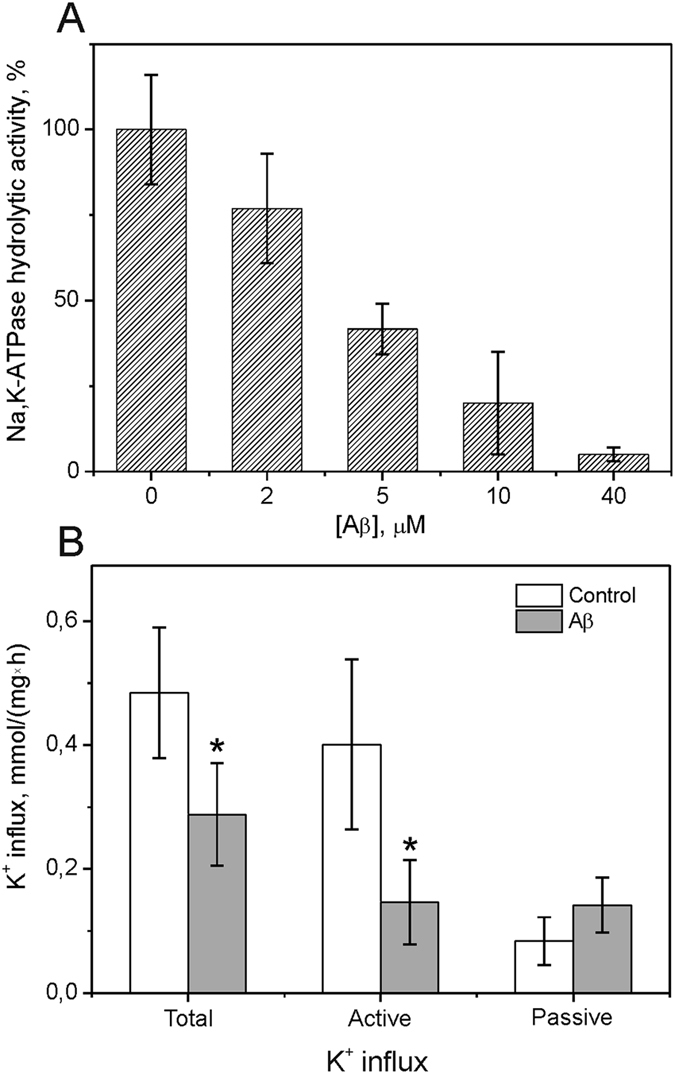
Effect of Aβ(1-42) on transport and hydrolytic activity of Na,K-ATPase in SH-SY5Y cells. (**A**) Hydrolytic activity of Na,K-ATPase after 30 min treatment of cells with different concentrations of Aβ(1-42). (**B**) K^+^(^86^Rb) influx (active influx reflects transport activity of Na,K-ATPase) in SH-SY5Y cells after 30 min treatment with 10 μM Aβ(1-42). Data are mean values for 4 independent experiments ± SE, *p < 0.04.

The inhibitory action of Aβ(1-42) on Na,K-ATPase was verified using neuroblastoma cells SH-SY5Y. The cells were incubated in the presence of Aβ(1-42) for 30 min. Then cells were lysed and hydrolytic activity of Na,K-ATPase in the lysates was measured (Fig. 2A). At concentration of Aβ(1-42) 40 μM inhibition of the enzyme activity reached 95% and the IC_50_ value is equal 4 ± 2 μM which correlates well with the data on the inhibition of the purified Na,K-ATPase preparation (Fig. 1B).

Transport activity of Na-K-ATPase was evaluated in intact cells using ^86^Rb as a radioactive tracer for K^+^. Pre-treatment by 10 μM Aβ(1-42) with or without 100 μM ouabain was followed by the addition of ^86^Rb^+^ to the cells (see Methods). As shown in Fig. 2B, total K^+^ influx in the cells treated with Aβ(1-42) was suppressed by 36% compared to the control values. Whereas active K^+^ influx, caused by Na,K-ATPase activity, was suppressed by 65%, the passive ouabain insensitive component (passive K^+^ influx) was unaltered by Aβ(1-42). Thus, introduction of Aβ(1-42) to the extracellular medium resulted in the inhibition of Na,K-ATPase transport activity in the cell.

### Effect of Aβ(1-42) on cell viability

Parameters characterizing viability of SH-SY5Y cells after 30 min incubation with Aβ(1-42) have been assessed by flow cytometry (percentage of apoptotic cells, cells with damaged membrane, the mitochondrial potential and level of intracellular calcium) and with xCelligence system allowing to evaluate the surface area occupied by cells on the plate. In the presence of 40 μM of Aβ(1-42) for 30 min, cell size was dramatically reduced (Supplementary Figure S2), the level of intracellular calcium fell by 71 ± 1%, however other parameters of cell viability remained unchanged (Supplementary Figures S3 and S4). This indicated that the cells were alive but ion homeostasis and osmoregulation were disrupted. At a concentration of 10 μM Aβ (1-42) did not affect the size and granularity of cells and the parameters of their viability after 30 min of incubation (Supplementary Figures S3 and S4). Differences in cell proliferation in the presence of 10 μM Aβ (1-42) became visible only after 15 h of incubation (Supplementary Figure S5).

### Aβ(1-42) penetration in neuroblastoma cells

**Figure 3:**
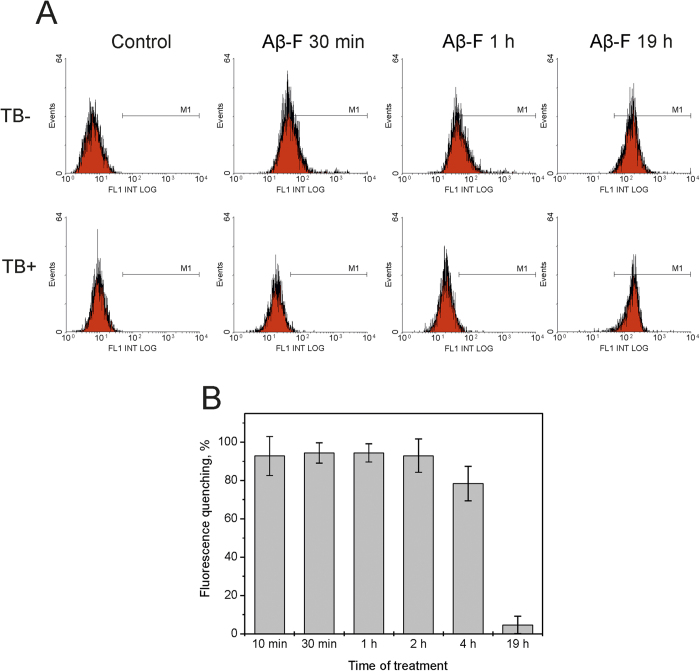
Penetration of fluorescein-Aβ(1-42) into SH-SY5Y cells. (**A**) Distribution of cells according to the intensity of green fluorescence after 30 min, 1 h and 19 h incubation with Aβ(1-42) labeled with fluorescein (Aβ-F) before (upper panels) and after (lower panels) addition of trypane blue (TB). (**B**) Quenching of cell fluorescence by trypane blue versus time of treatment by Aβ-F.

The ability of Aβ(1-42) to penetrate the SH-SY5Y cells was tested as they were treated by 10 μM of Aβ(1-42), labeled with fluorescein (fluorescein-Aβ(1-42)). We used trypan blue for quenching the extracellular peptide fluorescence^14^. Changes in the cell fluorescence intensity representing the amount of fluorescein-Aβ(1-42) bound to the cell surface and/or penetrated into the cells, relative to incubation time are presented in Fig. 3A. The fluorescence intensity of cells rises within one hour after treatment by fluorescein-Aβ(1-42). In the presence of trypan blue this fluorescence is almost totally quenched, i.e. fluorescein-Aβ(1-42) is located on the cell surface and does not penetrate into cells (Fig. 3B). Decrease in the effect of fluorescence quenching is observed initially after 4-hour incubation, indicating that the peptide has partially penetrated into cells and is no longer accessible for trypan blue (Fig. 3B). After 19 hours of the cells incubation with fluorescein-Aβ(1-42), fluorescence quenching by trypan blue is no longer observed, indicating intracellular location for the whole peptide sample labeled with fluorescein.

### Structure of the Aβ(1-42):Na,K-ATPase complex

**Figure 4:**
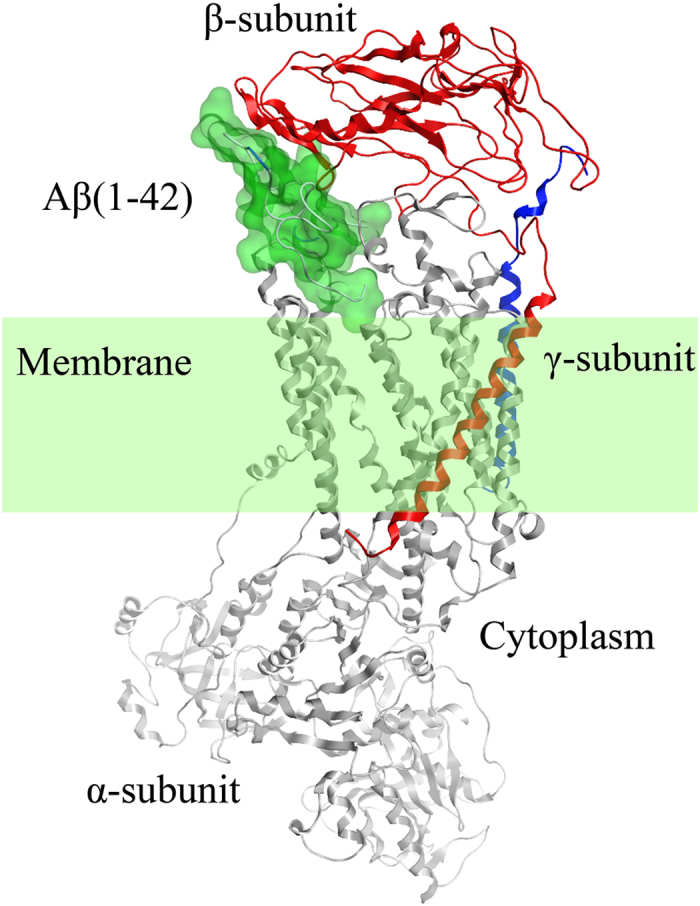
The model of Aβ(1-42):Na,K-ATPase complex. 3D model of the complex constructed on the basis of shark Na,K-ATPase α1β1 isozyme (PDB code 2zxe). The modeled Aβ(1-42) was docked to the protein using VinaAutoDock program^15^ (for details see Methods). Na,K-ATPase α-subunit is represented in gray, β-subunit in red, γ-subunit in blue. Aβ (1-42) is shown as translucent green molecular surface. Cell membrane is represented in translucent light green.

Model of the Aβ(1-42):Na,K-ATPase complex has been built using the shark Na,K-ATPase crystal structure (PDB code 2zxe) and Aβ(1-42) modeled structure. In all 9 top models of the complex generated by docking^15^, Aβ(1-42) was localized within the same binding site between the extracellular parts of the alpha- and beta-subunits of Na,K-ATPase (Supplementary Figure S6). The best score model is shown in Fig. 4. Pro125, Glu909, and Arg979 of the alpha-subunit and Glu88, Ser101, Glu273 and Arg292 of the beta-subunit form hydrogen and ionic bonds with Aβ(1-42) (Supplementary Figure S7). Electrostatic interactions in the Aβ(1-42)-Na,K-ATPase interface are in agreement with the enthalpy changes observed by ITC when the complex is formed. Aβ(1-42) residues are in close proximity (distance < 4.5 Å) of amino acid residues in the alpha and beta subunits (Supplementary Figure S7).

### Activation of Src-kinase by Aβ(1-42)

**Figure 5:**
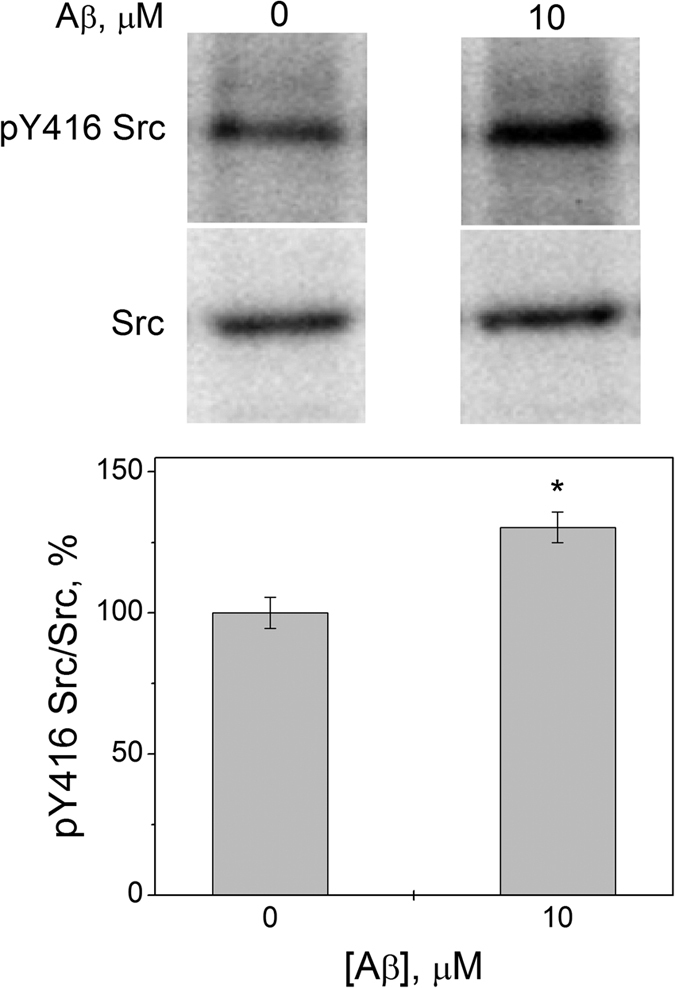
Activation of Src-kinase by Aβ(1-42). After 30 min of incubation with 10 μM Aβ(1-42) SH-SY5Y cells were lysed, and isolated proteins were separated by SDS-PAGE and probed in a Western blot by antibodies against total Src-kinase (Src) or phosphorylated Y416 Src-kinase (pY416 Src). Bars represent the changes in phosphorylation of Src-kinase normalized by its total amount (pY416 Src/Src). n = 3, mean ± SD; *p < 0.05. Shown above are the original immunoblotting readouts.

It is known that Na,K-ATPase interacts with Src-kinase to form a receptor complex^16^. To reveal the effect of Aβ(1-42) on receptor function of Na,K-ATPase, we have incubated SH-SY5Y cells with 10 μM of Aβ(1-42) for 30 min and measured the level of phosphorylation of the Src-kinase residue Tyr416. Autophosphorylation of Tyr416, which leads to activation of Src-kinase, was described for Src-kinase dissociation from the complex with Na,K-ATPase when binding ouabain^16^. We have found that incubation with Aβ(1-42) leads to an over 30% increase of phosphorylation level of the Src-kinase residue Tyr416 (Fig. 5). Thus binding of Aβ(1-42) to Na, K-ATPase, by analogy with ouabain leads to the activation of Src-kinase.

## Discussion

Decrease of Na,K-ATPase activity is one of the early markers of AD^2^,^17^,^18^,^19^,^20^. Na,K-ATPase plays a key role in maintaining ion balance and resting potential and hence is important for the regulation of neuronal excitability, and synaptic transmission. It is also involved in memory formation, possibly due to the effect on synaptic plasticity of neurons and long-term potentiation^3^,^5^. There are numerous data indicating that the decrease in activity of Na,K-ATPase is not a consequence of neurodegenerative processes but, on the contrary, precedes them and causes the disruption of neuronal activity, deregulation of cellular ion homeostasis, calcium overload and neuronal death^2^,^3^,^5^,^17^,^21^,^22^. On the other hand, there exists evidence that Aβ is required for the regulation of synaptic transmission^7^. We hypothesized that the physiological role of Aβ may involve regulation of the receptor and transport functions of Na,K-ATPase.

We have shown that Aβ(1-42) formed an equimolar complex with Na,K-ATPase that was unable to cleave ATP and transport ions (Figs 1 and 2). Inhibition of the Na,K-ATPase function in intact cells by Aβ(1-42) occurred within 30 minutes (Fig. 2B), whereas penetration of the Aβ(1-42) into the cells could be detected much later in time (Fig. 3). This observation suggested that the inhibitory effect is mediated by binding of the Aβ(1-42) to the extracellular domain of the enzyme. In line with these data, the model of the Aβ(1-42):Na,K-ATPase complex (Fig. 4) implied binding of the peptide to the extracellular part of Na,K-ATPase, in the “gap” between the alpha- and beta- subunits. According to our model, binding Aβ(1-42) hinders the movement of the alpha- and beta-subunit towards each other during the catalytic cycle^23^, disrupting the Na,K-ATPase function. Hence Aβ is a potential physiological endogenic regulator of Na,K-ATPase. Observed acute effects cannot be directly translated to the pathogenesis of Alzheimer’s disease associated with chronic effects of the Aβ.

The cardiotonic steroids (CTS) are known as endogenous regulators of Na,K-ATPase, which bind to the extracellular enzyme domains affecting its activity and receptor function^24^,^25^. K_d_ value for the Aβ(1-42) complex with Na,K-ATPase (3 μM) is close to the K_d_ for complexes of Na,K-ATPase with such CTS as ouabain (0.8 μM) and marinobufagenin (1.5 μM) at the same conditions^26^. CTS and Aβ are present in the blood plasma in similar concentrations, within several nanomoles^27^. Taking into account that binding of CTS to Na,K-ATPase triggers activation of Src-kinase^28^, it is reasonable to suggest that binding of Aβ to Na,K-ATPase may also cause the activation of the Src signaling pathway. Indeed, we have shown that incubation of neuroblastoma cells with Ab, leading to decrease of Na,K-ATPase activity, causes the activation of Src-kinase (Fig. 5). Previously, it was shown that treatment of neuronal cells by Aβ(1-42) leads to phosphorylation of a number of proteins within 10 minutes, and that this phosphorylation was blocked by addition of the Src family tyrosine kinase inhibitor^29^. Thus, it is tempting to suggest that physiological function of CTS and Aβ as signaling entities, as well as toxicity of abnormally high doses of both messengers, may share certain molecular mechanisms.

Endogenous CTS level varies in different pathologies, including ischemia^30^ and hypertension^24^, leading to the modulation of Na,K-ATPase activity and launching several signaling cascades in cells. There is only limited data on the causes of changes in the physiological levels of Aβ. It has been shown that the level of Aβ rises during stress due to the increase of APP processing^31^. It also rises upon the changes in hormonal status, particularly in the level of luteinizing hormone and testosterone^32^, and as a result of injuries^33^. Short-term reduction in Na,K-ATPase activity may allow cells to maintain the ATP pool and promote their viability^11^. Thus, short-term increase in the level of Aβ in the extracellular space and subsequent binding of Aβ to Na,K-ATPase would allow to regulate the activity of neuronal Na,K-ATPase under pathological conditions. Binding of Aβ would induce a number of signaling cascades increasing the adaptive capacity of the cells. As we have shown Aβ(1-42) in the 10 μM concentration decreases transport activity in the intact cells by 65% (Fig. 2), but has no toxic effect on cells in short incubation periods (Supplementary Figures S3 and S4) though it causes Src-kinase activation (Fig. 5). Even at the Aβ(1-42) concentration 40 μM, which completely inactivates Na,K-ATPase, the fraction of dead neuroblastoma cells in the population did not increase, although a disruption of ionic homeostasis and osmoregulation was observed (Supplementary Figures S2–S4). Exposure to Aβ(1-42) for longer periods of time led to the loss of viability of SH-SY5Y cells (Supplementary Figure S5), and, as we showed earlier, of the human differentiated neuronal cells^9^ and neuroblastoma cells SK-N-SH^34^. Hence, disruption of the Aβ homeostasis leading to a prolonged increase in Aβ local concentrations, e.g. in the synaptic cleft, will lead to the inhibition of Na,K-ATPase, imbalance of the Na^+^ and Ca^2+^ levels and the loss of neuronal excitability. Local concentrations of the peptide can vary widely and must reach high values before the aggregates and β-amyloid plaques will form. It was shown that at 1 nM concentration of Aβ(1-42) in the extracellular fluid, its accumulation was observed in endosomes/vesicles of SH-SY5Y cells after 24 h incubation^35^. At the concentration of 25 nM in extracellular fluid, the Aβ(1-42) concentration in vesicles constituted over 2.5 μM after 24 h incubation, i.e. a similar concentration was also reached at the cell surface^35^. These data imply that impaired homeostasis of β-amyloid will lead to its accumulation at cell surface creating high local concentrations of the peptide in the area of Na,K-ATPase localization even at the nanomolar concentrations of Aβ(1-42) in extracellular fluid. This is consistent with the hypothesis that disruption of the electrotonic properties of neurons observed in AD is a direct consequence of the long-term inhibition of Na,K-ATPase at early stages of the disease. Additionally, beta-subunit of the enzyme is involved in intercellular adhesion^36^, and Aβ binding to the extracellular part of Na,K-ATPase may lead to disruption of these contacts and to neuronal degeneration.

To summarize, we have shown that the monomeric Aβ(1-42) forms equimolar complex with the Na,K-ATPase, leading to acute inhibition of the enzyme transport and hydrolytic functions. Formation of Ab-ATPase complexes is therefore of high physiological importance. For example, short-term increase of Aβ(1-42) level under stress conditions^31^,^32^,^33^ will lead to reversible inhibition of Na,K-ATPase which could prevent ATP depletion and increasing cell viability. We have demonstrated that acute suppression of the Na,K-ATPase in intact neuroblastoma cells SH-SY5Y by Aβ(1-42) is caused by binding of the peptide to the extracellular part of the protein. Activation of Src-kinase in neuroblastoma cells indicates that Aβ(1-42) is a potential activator of the Na,K-ATPase-mediated signal transduction. Our data contribute to the understanding of the physiological role of Aβ, acting as a regulator of the functional activity of Na,K-ATPase.

## Methods

### Na,K-ATPase and Aβ(1-42) preparations

Synthetic peptide Aβ(1-42): [H2N]-DAEFRHDSGYEVHHQKLVFFAEDVGSNKGAIIGLMVGGVVIA-[COOH] was purchased from Biopeptide. Preparation of the monomeric form of Aβ(1-42) was performed as described elsewhere^37^. To do this, cold hexafluoroisopropanol (Fluka) was added to dry Aβ (1-42) to a concentration of 1 mM and incubated for 60 min at room temperature. Then this solution was put on ice for 10 min and aliquoted into non-siliconized microcentrifuge tubes (0.56 mg peptide per tube). Peptide in the tubes was dried under vacuum using Eppendorf Concentrator 5301. Dried peptide was stored at −80 °C. 2.5 mM peptide stock solution was prepared by adding 20 μL of 100% anhydrous DMSO (Sigma-Aldrich) to 0.22 mg peptide and incubating for 1 h at room temperature. For use in the experiments, the peptide was diluted to the required concentration with buffer solution. Equivalent amount of DMSO was added to the control samples in all experiments. Only freshly prepared peptide solutions were used for all experiments. By dynamic light scattering (DLS) and turbidity methods it was shown that there were no aggregates and higher molecular weight oligomers in Aβ(1-42) preparation (40 μM) after 1 hour following preparation of the water solution. To determine the turbidity the optical density at 405 nm of freshly prepared solutions of Aβ(1-42) was recorded on Jasco V-560 spectrophotometer within one hour. The absorbance of the solution does not change, which allows to make a conclusion about the absence of particles in solution with a size of 1–100 nm. DLS method allows measuring the particle size from 0.6 to 10 nm. The DLS measurements were carried out on a Zetasizer Nano ZS apparatus (Malvern Instruments Ltd.). According to our data the freshly prepared solution of amyloid (40 μM) does not contain particles in this size range.

The monomer and low molecular weight oligomer quantities in Aβ(1-42) solution were estimated by SDS-PAGE with pre-stabilization of oligomers by photoinduced crosslinking using covalent Tris (2,2-bipyridyl) dichlororuthenium (II) hexahydrate^38^. The monomers constituted 80% in Aβ(1-42) preparation (Supplementary Figure S8).

Purified preparation of Na,K-ATPase (α1β1 isozyme) was obtained from duck salt glands as described elsewhere^11^,^39^. The purity grade of Na,K-ATPase was 99% (Supplementary Figure S9) and specific activity of the enzyme reached ~2400 μmol of Pi (mg of protein × h)^−1^ at 37 °C.

### Cell culture

Undifferentiated human neuroblastoma SH-SY5Y cells were cultured in DMEM/F-12 media, containing 20% fetal bovine serum (FBS), 0.2 mg/ml Penicillin-Streptomycin, and 0.01 mM sodium pyruvate on uncoated petri dishes at 37 °C in humid atmosphere with 5% CO_2_. Notably, non-differentiated SH-SY5Y cells are a ubiquitous model frequently used to study mechanism of the Аβ(1-42) effects on cells^35^,^40^. Amount of Na,K-ATPase at the SH-SY5Y cell surface was close to the amount present at the surface of primary neuronal granular cells, as confirmed be similar values of Na,K-ATPase transport activity in these cells (active K^+^ influx 0.40 mM/(mg × h) for SH-SY5Y cells, Figure 2B, versus 0.35 mM/(mg × h) for primary neurons^41^). Cell lysates containing Na,K-ATPase were prepared by repeated freezing-thawing cycles^41^.

### Hydrolytic and transport Na,K-ATPase activity measurements

Hydrolytic activity of Na,K-ATPase in the purified preparation and in cell lysates was measured as ouabain-sensitive (1 mM) ATP cleavage in the medium containing 130 mM NaCl, 20 mM KCl, 3 mM MgCl_2_, 3 mM ATP, and 30 mM imidazole, pH 7.4, 37 °C as described in^41^,^42^,^43^.

Transport activity of the enzyme was measured in a separate set of experiments in intact cells using ^86^Rb as a radioactive tracer for K^+^. Ouabain-sensitive unidirectional K^+^(^86^Rb) influx was measured on the Petri dishes (diameter 3 cm) containing Tyrode solution. To distinguish between the active, Na,K-ATPase-mediated, and passive K^+^ influx, ouabain at the final concentration of 100 μM was added to one-half of the samples 15 min before the addition of the radioactive tracer. Aβ(1-42) was added 10 min before introduction of the radioactive tracer. Flux measurements were started by adding ^86^RbCl (~0.5 μCi/ml cell suspension; Perkin-Elmer). After 10–60 min of incubation with ^86^Rb, flux was stopped by immediate dilution with 10 ml ice-cold washing medium (100 mM Mg(NO_3_)_2_ and 10 mM imidazole, pH 7.4, 4 °C). After additional washing from external ^86^Rb cells were lysed in 5% TCA. Radioactivity of cells and incubation medium was measured using a Tri-Carb 1600 TR liquid scintillation counter (Packard) in water phase (Cherenkov effect). Accumulation of ^86^Rb^+^ by the neuroblastoma cells was linear within at least 30 min incubation with the tracer. Uptake over this time interval was used for the calculations of the unidirectional K^+^ influx. Unidirectional fluxes (J) were calculated using the following equation: J = (A_c_/A_m_ [K^+^]_e_)/*t*m_protein_, where A_c_ and A_m_ are radioactivity of cells in 1 ml suspension and 1 ml medium, respectively; m_protein_ is the amount of protein (mg/ml cell suspension) corrected for the amount of viable cells in suspension, [K^+^]_e_ is K^+^ concentration in the incubation medium, and *t* is the equilibration time with the tracer.

### Isothermal titration calorimetry (ITC)

The thermodynamic parameters of Aβ(1-42) binding to Na,K-ATPase were measured using a MicroCal iTC200 instrument, as described elsewhere^44^,^45^. Experiments were carried out at 25 °C in 10 мМ imidazole buffer (pH 7.5), containing 130 мМ NaCl, 30 мM KCl, 3 мМ MgCl_2_. Aliquots (2.6 μl) of ligands were injected into a 0.2-ml cell containing protein solution to achieve a complete binding isotherm. Protein concentration in the cell ranged from 5 to 20 μM, and ligand concentration in the syringe ranged from 50 to 200 μM. The resulting titration curves were fitted using the MicroCal Origin software, assuming one set of binding sites. Affinity constants (K_a_), enthalpy variations (ΔH) and stoichiometry of binding (N) were determined and the Gibbs energy (ΔG) and entropy variations (ΔS) were calculated from the equation: ΔG = -RTlnKa = ΔH-TΔS.

### Aβ(1-42) labeling and monitoring its penetration into cells

Aβ(1-42) was labeled by NHS495 dye (amine-reactive derivative of fluorescein, E_ex_/E_em_ = 488/535 нм). Six microliters of Aβ(1-42) (2.5 mM) mixed with 15 μl of NHS495 (1 mM) were incubated one hour at room temperature. To remove the unreacted “free” dye the volume of reaction mixture was adjusted to 500 μL by phosphate buffer, pH 7.4 and then passed through desalting column PD MidiTrap G-25 (GE Healthcare). Labeled Aβ(1-42) was added to the cell suspension at a concentration of 10 μM. Penetration of Aβ(1-42) into cells was evaluated by flow cytometry on GALLIOS flow cytometer (Beckman Coulter). The distinction between internalized and surface-bound fluorescein-Aβ(1-42) was performed using trypan blue (1.2 mg/ml) for quenching surface-bound fluorescence as described in^14^. The fluorescence of living cells was assessed after 10, 30, 60, 120, 180 min and 19 h with or without trypan blue.

### Flow cytometry

Analysis of the cells was performed on GALLIOS flow cytometer (BeckmanCoulter). In our analysis we have excluded cell debris lying outside the gate R1 (Supplementary Figure S2). The size of the cells was assessed by forward side scatter (FS). Cells with damaged membrane were determined according to staining with propidium iodide (PI) (Sigma) (Ex/Em = 535/617 nm). Mitochondrial membrane potential in intact cells (Ψ) was detected by MitoProbe DilC1(5) (Ex/Em = 638/658 nm) (Invitrogen), according to Mironova *et al*.^46^. The Ca^2+^ level in intact cells was estimated by staining with fluo-4 (Ex/Em 494/516 nm) (Molecular Probes), according to Mitkevich *et al*.^47^. Each value is the mean of at least three independent experiments with triplicate samples ± SD.

### Immunoblot analysis

Cells were incubated with 10 μM Aβ(1-42) for 30 min and then lysed in the RIPA buffer (25 mM tris-HCl, pH 7.6, 150 mМ NaCl, 1% Nonidet-P40, 0.1% SDS, 1% sodium deoxycholate) containing 1 μM of PMSF with stirring at 4 °C for 1 h. The probes were then centrifuged at 13000 g for 10 min and the supernatant was collected. Proteins of cell lysates were separated on SDS-PAGE and transferred to a PVDF membrane. After membrane blocking in 5% nonfat milk in PBST, the detection of phospho (Tyr 416) Src and total Src was carried out by incubating the membrane in the solution of appropriate rabbit polyclonal antibodies (both from Cell Signaling Technology) in PBST. The level of β-actin was also estimated using mouse monoclonal anti-β-actin antibody (Ambion). Visualization of the proteins was performed by the appropriate horseradish peroxidase-conjugated secondary antibodies provided by the enhanced chemiluminescence SuperSignal ™ West Femto Maximum Sensitivity Substrate kit (ThermoScientific). Chemiluminescence was detected using Bio-Rad ChemiDoc MP instrument. Densitometric analysis was performed with Image Lab program (Bio-Rad) and the results were expressed as ratio of phospho-Src to total Src band intensity (phospho-Src/Src).

### Statistical analysis

The comparison of data groups was performed using Student’s t-test; p < 0.05 was considered significant. ITC, hydrolytic activity and flow cytometry data are presented as means of at least three independent experiments ± SD. Data of K^+^(^86^Rb) influx are presented as means of four independent experiments ± SE.

### Modeling of the structure of Aβ(1-42):Na,K-ATPase complex

Model of the Aβ(1-42) peptide was constructed using as templates the Aβ(1-16) structures 1ze7 and 4f37 (PDB ids) and the *ab initio* model of Aβ(1-42) built using the server Bhageerath^48^. The resulting model of Aβ(1-42) was minimized in the AMBER99 force field with the MOE program version 2013.08. Modeling of the Aβ(1-42):Na,K-ATPase complex was performed using the structure of Na,K-ATPase from shark glands 2zxe (PDB id) solved at 2.4 Å resolution^49^, and the modeled structure of Aβ(1-42). Docking has been carried out with VinaAutoDock program^15^, and the docking was constrained to cover only the extracellular part of the protein.

### Determination of Cell index

Analysis was performed on xCELLigence real time cell analyser (RTCA) (ACEA Biosciences) measures focal adhesion of living cells in real-time^50^. Cells were seeded onto custom RTCA E-plates (ACEA Biosciences) coated with high-density gold arrays for measuring electrical impedance. The xCELLigence biosensor measures cellular adhesion, which is converted to Cell index (unit less) by the xCELLigence software (version 1.2.1). Cells were seeded into E16 well plates and allowed to recover until the cells had attained a stable Cell index. Treatment of cells on the plates with Aβ(1-42) was performed in serum free DMEM. After 4 h of treatment inactivated FBS (10%) was added into plates.

## Additional Information

**How to cite this article**: Petrushanko, I. Yu. *et al*. Direct interaction of beta-amyloid with Na,K-ATPase as a putative regulator of the enzyme function. *Sci. Rep*. **6**, 27738; doi: 10.1038/srep27738 (2016).

## Supplementary Material

Supplementary Information

## References

[b1] Boutilier R. G. Mechanisms of cell survival in hypoxia and hypothermia. J Exp Biol 204, 3171–3181 (2001). 11581331 10.1242/jeb.204.18.3171

[b2] Dickey C. A. . Dysregulation of Na^+^/K^+^ ATPase by amyloid in APP+PS1 transgenic mice. BMC Neurosci. 2, 1–7 (2005). 10.1186/1471-2202-6-7 PMC549198 15689237

[b3] Zhang L. N. . Na^+^ -K^+^ -ATPase, a potent neuroprotective modulator against Alzheimer disease. Fundam. Clin. Pharmacol. 27, 96–103 (2013). 23033963 10.1111/fcp.12000

[b4] Kairane C., Mahlapuu R., Ehrlich K., Zilmer M. & Soomets U. The effects of different antioxidants on the activity of cerebrocortical MnSOD and Na,K-ATPase from post mortem Alzheimer’s disease and age-matched normal brains. Curr. Alzheimer Res. 11, 79–85 (2014). 24156257 10.2174/15672050113106660179

[b5] Kreutz F. . Alterations on Na^+^, K^+^ -ATPase and acetylcholinesterase activities induced by amyloid-β peptide in rat brain and GM1 ganglioside neuroprotective action. Neurochem Res 38, 2342–2350 (2013). 24013887 10.1007/s11064-013-1145-6

[b6] Kang J. . The precursor of Alzheimer’s disease amyloid A4 protein resembles a cell surface receptor. Nature 325, 733–736 (1987). 2881207 10.1038/325733a0

[b7] Gregory G. C., Shepherd C. E. & Halliday G. M. Physiologic and Neurotoxic Properties of Aβ Peptides. In eds Barrow C. J. , Small D.H. Abeta Peptide and Alzheimer’s Disease: Celebrating a Century of Research. London, Springer-Verlag, 308 pp (2007).

[b8] Garcia-Osta A. & Alberini C. M. Amyloid beta mediates memory formation. Learn. Mem. 16, 267–272 (2009). 19318468 10.1101/lm.1310209 PMC2661754

[b9] Mitkevich V. A. . Isomerization of Asp7 leads to increased toxic effect of amyloid- β42 on human neuronal cells. Cell Death & Disease 4, e939 (2013). 24287700 10.1038/cddis.2013.492 PMC3847340

[b10] Ohnishi T. . Na, K-ATPase α3 is a death target of Alzheimer patient amyloid-β assembly. Proc Natl Acad Sci USA 112, E4465–E4474 (2015). 26224839 10.1073/pnas.1421182112 PMC4538662

[b11] Petrushanko I. Y. . S-glutathionylation of the Na,K-ATPase catalytic α subunit is a determinant of the enzyme redox sensitivity. J. Biol. Chem. 287, 32195–32205 (2012). 22798075 10.1074/jbc.M112.391094 PMC3442550

[b12] O’Brien R. & Hag I. Biocalorimetry 2. Applications Calorimetry In The Biological Sciences. (eds. Ladbury J. E. & Doyle M. L.) Ch. 1, 3–34 (The Sussex John Wiley & Sons, Ltd, 2004).

[b13] Paula S., Tabet M. R. & Ball W. J. J. Interactions between cardiac glycosides and sodium/potassium-ATPase: three-dimensional structure-activity relationship models for ligand binding to the E2-Pi form of the enzyme versus activity inhibition. Biochemistry 44, 498–510 (2005). 15641774 10.1021/bi048680w

[b14] Nuutila J. & Lilius E. M. Flow cytometric quantitative determination of ingestion by phagocytes needs the distinguishing of overlapping populations of binding and ingesting cells. Cytometry A 65, 93–102 (2005). 15825183 10.1002/cyto.a.20139

[b15] Trott O. & Olson A. J. AutoDock Vina: improving the speed and accuracy of docking with a new scoring function, efficient optimization, and multithreading. J Comput Chem. 31, 455–461 (2010). 19499576 10.1002/jcc.21334 PMC3041641

[b16] Banerjee M., Duan Q. & Xie Z. SH2 ligand-like effects of second cytosolic domain of Na/K-ATPase α1 subunit on Src kinase. PLoS One 10, e0142119 (2015). 26551526 10.1371/journal.pone.0142119 PMC4638348

[b17] Mark R. J., Hensley K., Butterfield D. A. & Mattson M. P. Amyloid beta-peptide impairs ion-motive ATPase activities: evidence for a role in loss of neuronal Ca2+ homeostasis and cell death. J Neurosci. 15, 6239–6249 (1995). 7666206 10.1523/JNEUROSCI.15-09-06239.1995 PMC6577674

[b18] Vitvitsky V. M., Garg S. K., Keep R. F., Albin R. L. & Banerjee R. Na+ and K+ ion imbalances in Alzheimer’s disease. Biochim Biophys Acta 1822, 1671–1681 (2012). 22820549 10.1016/j.bbadis.2012.07.004 PMC3444663

[b19] Hattori N. . CI-ATPase and Na^+^/K^(+)^-ATPase activities in Alzheimer’s disease brains. Neurosci Lett 254, 141–144 (1998). 10214977 10.1016/s0304-3940(98)00654-5

[b20] Kairane C. . Regulation of the frontocortical sodium pump by Na^+^ in Alzheimer’s disease: difference from the age-matched control but similarity to the rat model. FEBS Lett. 531, 241–244 (2002). 12417319 10.1016/s0014-5793(02)03510-x

[b21] Gu Q. B., Zhao J. X., Feib J. & Sshwarz W. Modulation of Na,K pumping and neurotransmitter uptake by b-amyloid. Neuroscience 126, 61–67 (2004). 15145073 10.1016/j.neuroscience.2004.03.022

[b22] Pákáski M. & Kálmán J. Interactions between the amyloid and cholinergic mechanisms in Alzheimer’s disease. Neurochem Int 53, 103–111 (2008). 18602955 10.1016/j.neuint.2008.06.005

[b23] Dempski R. E., Hartung K., Friedrich T. & Bamberg E. Fluorometric measurements of intermolecular distances between the alpha- and beta-subunits of the Na^+^/K^+^-ATPase. J Biol Chem 281, 36338–36346 (2006). 16980302 10.1074/jbc.M604788200

[b24] Schoner W. & Scheiner-Bobis G. Endogenous and exogenous cardiac glycosides and their mechanisms of action. Am. J. Cardiovasc. Drugs 7, 173–189 (2007). 17610345 10.2165/00129784-200707030-00004

[b25] Bagrov A. Y. & Fedorova O. V. Effects of two putative endogenous digitalis-like factors, marinobufagenin and ouabain, on the Na^+^, K^+^-pump in human mesenteric arteries. J Hypertens. 16, 1953–1958 (1998). 9886882 10.1097/00004872-199816121-00015

[b26] Klimanova E. A. . Binding of ouabain and marinobufagenin leads to different structural changes in Na,K-ATPase and depends on the enzyme conformation. FEBS Lett 589, 2668–2674 (2015). 26297827 10.1016/j.febslet.2015.08.011

[b27] Bagrov A. Y., Shapiro J. I. & Fedorova O. V. Endogenous cardiotonic steroids: physiology, pharmacology, and novel therapeutic targets. Pharmacol Rev. 61, 9–38 (2009). 19325075 10.1124/pr.108.000711 PMC2763610

[b28] Li Z. & Xie Z. J. The Na/K-ATPase/Src complex and cardiotonic steroid-activated protein kinase cascades. Pflugers Arch. 457, 635–644 (2009). 18283487 10.1007/s00424-008-0470-0

[b29] Williamson R. . apid tyrosine phosphorylation of neuronal proteins including tau and focal adhesion kinase in response to amyloid-beta peptide exposure: involvement of Src family protein kinases. J Neurosci 22, 10–20 (2002). 11756483 10.1523/JNEUROSCI.22-01-00010.2002 PMC6757621

[b30] Bagrov A. Y. . Characterization of a urinary bufodienolide Na^+^,K^+^-ATPase inhibitor in patients after acute myocardial infarction. Hypertension 31, 1097–1103 (1998). 9576120 10.1161/01.hyp.31.5.1097

[b31] Marcello E., Gardoni F. & Di Luca M. Alzheimer’s disease and modern lifestyle: what is the role of stress? J Neurochem 134, 795–798 (2015). 26206000 10.1111/jnc.13210

[b32] Verdile G. . The impact of luteinizing hormone and testosterone on beta amyloid (Aβ) accumulation: Animal and human clinical studies. Horm Behav, doi: 10.1016/j.yhbeh.2015.05.020 (2015). 26122291

[b33] Justice N. J. . Posttraumatic stress disorder-like induction elevates β-amyloid levels, which directly activates corticotropin-releasing factor neurons to exacerbate stress responses. J Neurosci 35, 2612–2623 (2015). 25673853 10.1523/JNEUROSCI.3333-14.2015 PMC4323535

[b34] Yurinskaya M. M. . Heat-shock protein HSP70 protects neuroblastoma cells SK-N-SH from the neurotoxic effects hydrogen peroxide and the β-amyloid peptide. Mol Biol (Moscow) 49, 1030–1034 (2015). 26710786 10.7868/S0026898415060233

[b35] Hu X. . Amyloid seeds formed by cellular uptake, concentration, and aggregation of the amyloid-beta peptide. Proc Natl Acad Sci USA 106, 20324–20329 (2009). 19910533 10.1073/pnas.0911281106 PMC2787156

[b36] Vagin O., Dada L. A., Tokhtaeva E. & Sachs G. The Na-K-ATPase α_1_β_1_ heterodimer as a cell adhesion molecule in epithelia. Am J Physiol Cell Physiol 302, C1271–C1281 (2012). 22277755 10.1152/ajpcell.00456.2011 PMC3361946

[b37] Klein W. L. Abeta toxicity in Alzheimer’s disease: globular oligomers (ADDLs) as new vaccine and drug targets. Neurochem Int. 41, 345–352 (2002). 12176077 10.1016/s0197-0186(02)00050-5

[b38] Rahimi F., Maiti P. & Bitan G. Photo-Induced Cross-Linking of Unmodified Proteins (PICUP) Applied to Amyloidogenic Peptides. Journal of visualized experiments 23, 1071 (2009). 19229175 10.3791/1071 PMC2763294

[b39] Smith T. W. Purification of Na^+^,K^+^-ATPase from the supraorbital salt gland of the duck. Methods Enzymol. 156, 46–48 (1988). 2835626 10.1016/0076-6879(88)56007-x

[b40] Kim Y. . The novel RAGE interactor PRAK is associated with autophagy signaling in Alzheimer’s disease pathogenesis. Mol Neurodegener 11, 4 (2016). 26758977 10.1186/s13024-016-0068-5 PMC4709948

[b41] Petrushanko I. . Na-K-ATPase in rat cerebellar granule cells is redox sensitive. Am. J. Physiol. Regul. Integr. Comp. Physiol. 290, R916–925 (2006). 16293684 10.1152/ajpregu.00038.2005

[b42] Komniski M. S., Yakushev S., Bogdanov N., Gassmann M. & Bogdanova A. Interventricular heterogeneity in rat heart responses to hypoxia: the tuning of glucose metabolism, ion gradients, and function. Am. J. Physiol. Heart Circ. Physiol. 300, H1645–1652 (2011). 21398597 10.1152/ajpheart.00220.2010

[b43] Rathbun W. B. & Betlach M. V. Estimation of enzymically produced orthophosphate in the presence of cysteine and adenosine triphosphate. Anal. Biochem. 28, 436–445 (1969). 5781434 10.1016/0003-2697(69)90198-5

[b44] Petrushanko I. Y. . Critical role of γ-phosphate in structural transition of Na,K-ATPase upon ATP binding. Scientific Reports 4, 5165 (2014). 24893715 10.1038/srep05165 PMC4044624

[b45] Mitkevich V. A. . GTPases IF2 and EF-G bind GDP and the SRL RNA in a mutually exclusive manner. Scientific Reports 2, 843 (2012). 23150791 10.1038/srep00843 PMC3496166

[b46] Mironova N. L. . Ribonuclease binase inhibits primary tumor growth and metastases via apoptosis induction in tumor cells. Cell Cycle 12, 2120–2131 (2013). 23759588 10.4161/cc.25164 PMC3737314

[b47] Mitkevich V. A. . Ribonuclease binase apoptotic signature in leukemic Kasumi-1 cells. Biochimie 95, 1344–1349 (2013). 23499289 10.1016/j.biochi.2013.02.016

[b48] Shenoy S. R. & Jayaram B. Proteins: sequence to structure and function–current status. Curr Protein Pept Sci 11 498–514 (2010). 20887265 10.2174/138920310794109094

[b49] Shinoda T., Ogawa H., Cornelius F. & Toyoshima C. Crystal structure of the sodium-potassium pump at 2.4 A resolution. Nature 459, 446–450 (2009). 19458722 10.1038/nature07939

[b50] Wiltshire R. . Regulation of human cerebro-microvascular endothelial baso-lateral adhesion and barrier function by S1P through dual involvement of S1P1 and S1P2 receptors. Scientific Reports 6, 19814 (2016). 26813587 10.1038/srep19814 PMC4728386

