# Supplementary material for: Direct interaction of beta-amyloid with Na,K-ATPase as a putative regulator of the enzyme function
Source: Sci Rep. 2016 Jun 14;6:27738. doi: 10.1038/srep27738 (PMC4906314; doi:10.1038/srep27738)
Supplement: Supplementary Information [file srep27738-s1.doc]

Supplementary materials

**Direct interaction of beta-amyloid with Na,K-ATPase as a putative regulator of the enzyme function**

Irina Yu. Petrushanko^1,#^, Vladimir A. Mitkevich^1,#^, Anastasia A. Anashkina^1^, Alexei A. Adzhubei^1^, Ksenia M. Burnysheva^1^, Valentina A. Lakunina^1^, Yulia V. Kamanina^1^, Elena A. Dergousova^1^, Olga D. Lopina^2^, Omolara O. Ogunshola^3^, Anna Yu. Bogdanova^3^, Alexander A. Makarov^1^,*

^1^Engelhardt Institute of Molecular Biology, Russian Academy of Sciences, Vavilov St. 32, 119991 Moscow, Russia

^2^Faculty of Biology, M.V. Lomonosov Moscow State University, 119234 Moscow, Russia

^3^Institute of Veterinary Physiology, Vetsuisse Faculty, and the Zurich Center for Integrative Human Physiology (ZIHP), University of Zurich, CH-8057 Zurich, Switzerland

^#^These authors contributed equally to this work

*To whom correspondence should be addressed: Alexander A. Makarov, Engelhardt Institute of Molecular Biology, RAS, Vavilov St. 32, 119991 Moscow, Russia, Phone: +7 499 1354095, Fax: +7 499 1351405, E-mail: [aamakarov@eimb.ru](mailto:aamakarov@eimb.ru)

Figure S1. Demonstration of the reversibility of Na,K-ATPase inhibition by Aβ(1-42). Samples with Na,K-ATPase from the duck salt glands, incubated for 30 minutes with 40 μM Aβ(1-42), were diluted 1:2, 1:4, and 1:10 respectively and hydrolytic activity of the enzyme was measured. The values are the average of three measurements ± SD; *p <0.001, **p <0.05

B

| A |  |
| --- | --- |
| I | II |
| **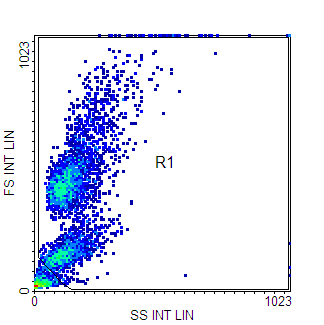** | **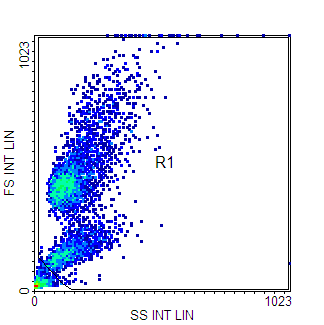** |
| III | IV |
| **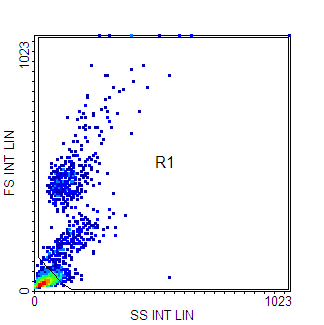** | **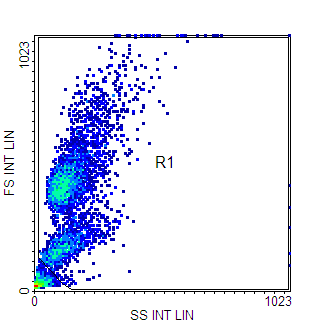** |
| B | C |
|  |  |

Figure S2. Changes in the average size of neuroblastoma SH-SY5Y cells resulting from exposure to 10 μM and 40 μM Aβ (1-42), and controls containing the corresponding amount of DMSO after 30 min of incubation. (A) Typical flow cytometry density plot of the forward (FS) and side (SS) scatter of cells describing size and granularity of SH-SY5Y cells, respectively. Cell population is marked by Gate R1. (I) 10 µM and (III) 40 µM of Aβ(1-42), (II) 0.4% and (IV) 1.6% DMSO; (B) Mean value of the FS parameter. (C) Change in Cell Index characterizing the surface area occupied by cells on the substrate after 30 min (dark gray columns) of incubation with DMSO and Aβ(1-42). The cell index before incubation (light gray columns) taken as a unit. The values are the average of three measurements ± SD; *p <0.05.

| A |  |
| --- | --- |
| I | II |
| 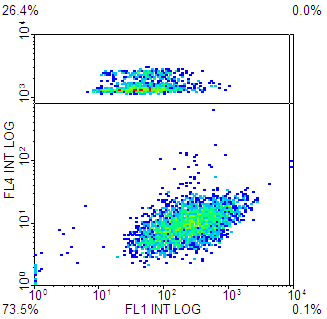 | 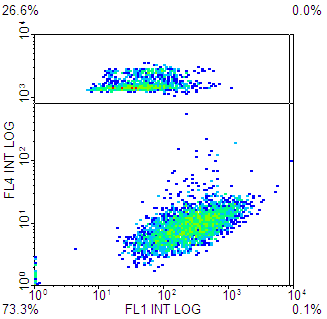 |
| III | IV |
| 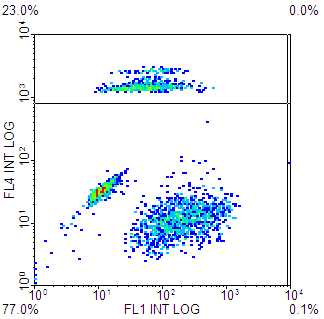 | 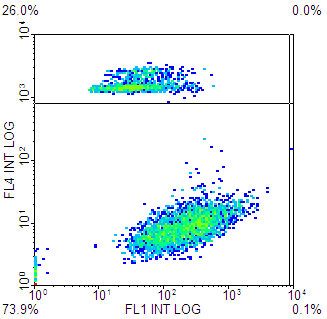 |
| B |  |
|  | |

Figure S3. Changes in intracellular calcium levels in neuroblastoma cells SH-SY5Y under the effect of 10 μM and 40 μM Aβ (1-42), and controls containing the corresponding amount of DMSO, after 30 min of incubation. (A) Typical flow cytometry density plot, describing the distribution of SH-SY5Y cells by dye fluorescence intensity sensitive to Ca^2+^ (FL1) and propidium iodide (FL4). (I) 10 μM and (III) 40 μM of Aβ (1-42), (II) 0.4% and (IV) 1.6% DMSO. (B) Mean value of Ca^2+^ level in the PI-negative (intact) cells. The values are the average of three measurements ± SD; *p <0.01, **p <0.001.

| A |  |
| --- | --- |
| I | II |
| 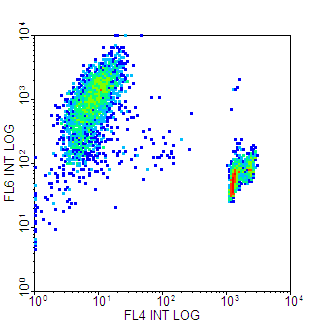 | 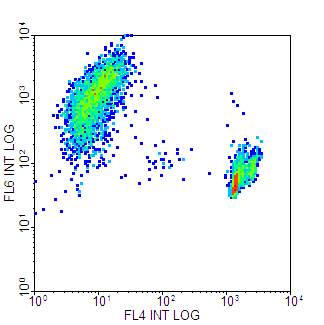 |
| III | IV |
| 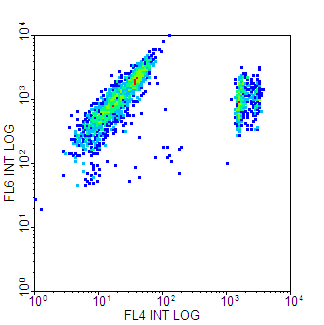 | 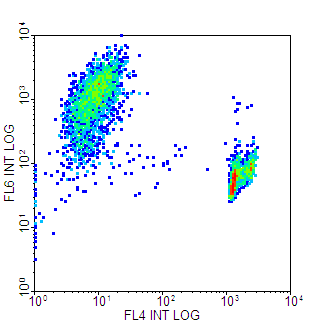 |
| B | C |
|  |  |

Figure S4. Changes in the percentage of neuroblastoma cells SH-SY5Y with the reduced mitochondrial potential (ψ) and damaged membrane (PI-positive sells) under the effect of 10 μM and 40 μM Aβ (1-42), and controls containing the corresponding amount of DMSO, after 30 min of incubation. (A) Typical flow cytometry density plot, describing the distribution of SH-SY5Y cells by fluorescence intensity of the DilC mitochondrial probe (FL6) and propidium iodide (FL4); (I) and 10 μM (III) 40 μM of Aβ (1-42), (II) 0.4% and (IV) 1.6% DMSO. (B) Percentage of intact neuroblastoma cells SH-SY5Y with reduced ψ and (C) the percentage of cells with damaged membrane. The values are the average of three measurements ± SD; *p <0.05.

Figure S5. Changes in Cell index for the neuroblastoma SH-SY5Y cells under the effect of the 10μM Aβ (1-42) during 24 hours of incubation. Red line denotes the cells treated with Aβ(1-42), control is shown by black line. The arrows mark time when Aβ(1-42) and fetal bovine serum (FBS) were added. The values are the average of four measurements ± SD.


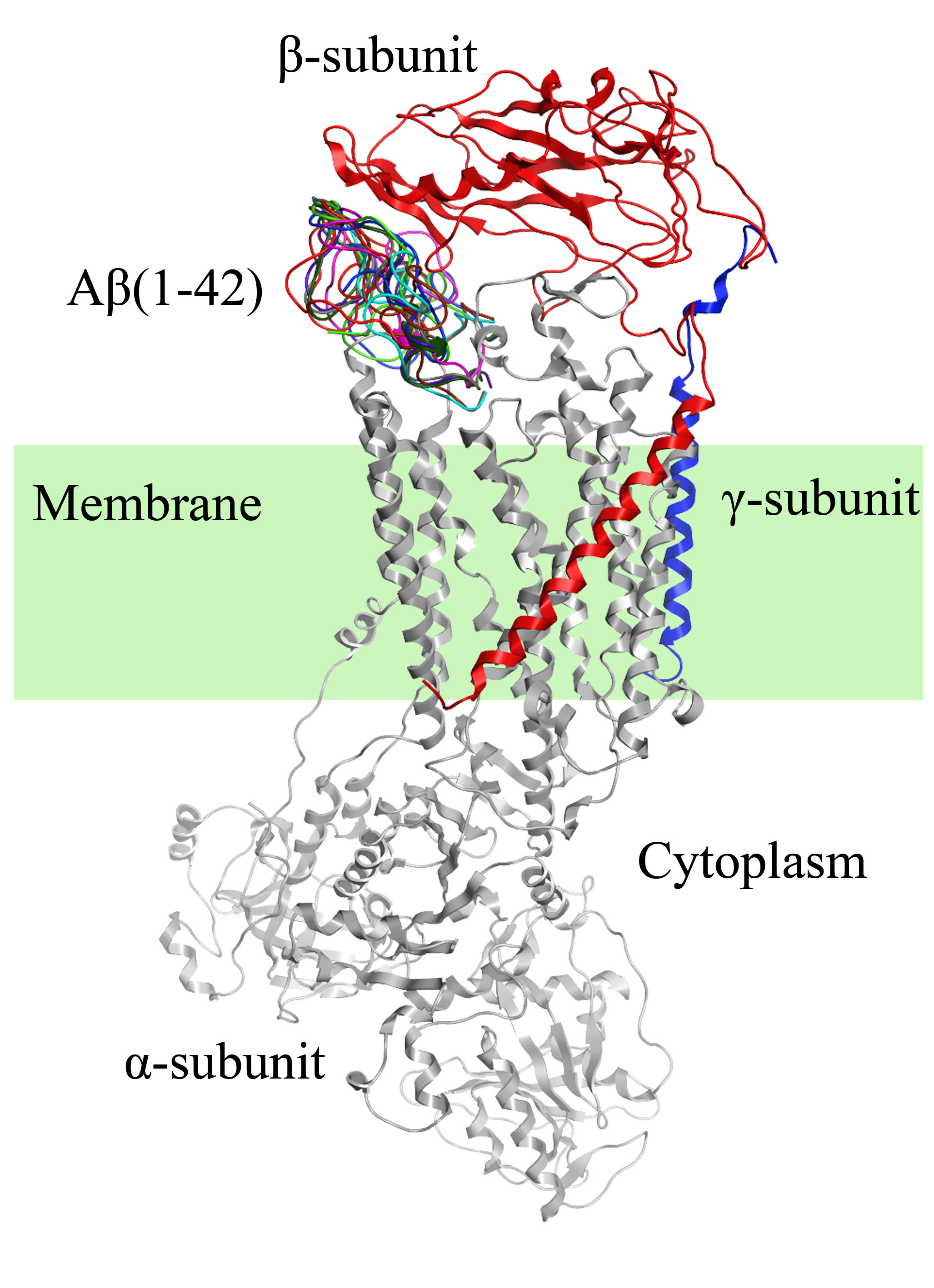


Figure S6. Aβ(1-42) docking to Na,K-ATPase. Nine possible locations of the Aβ(1-42) peptide ( shown as ribbons of different colors) in complex with Na,K-ATPase are presented. 3D model of the complex was constructed on the basis of shark Na,K-ATPase α1β1 isozyme structure (PDB code 2zxe). The modeled Aβ(1-42) was docked to the protein using VinaAutoDock program ^15^ (for details see Methods). Na,K-ATPase α-subunit is represented in gray, β-subunit in red, γ-subunit in blue.


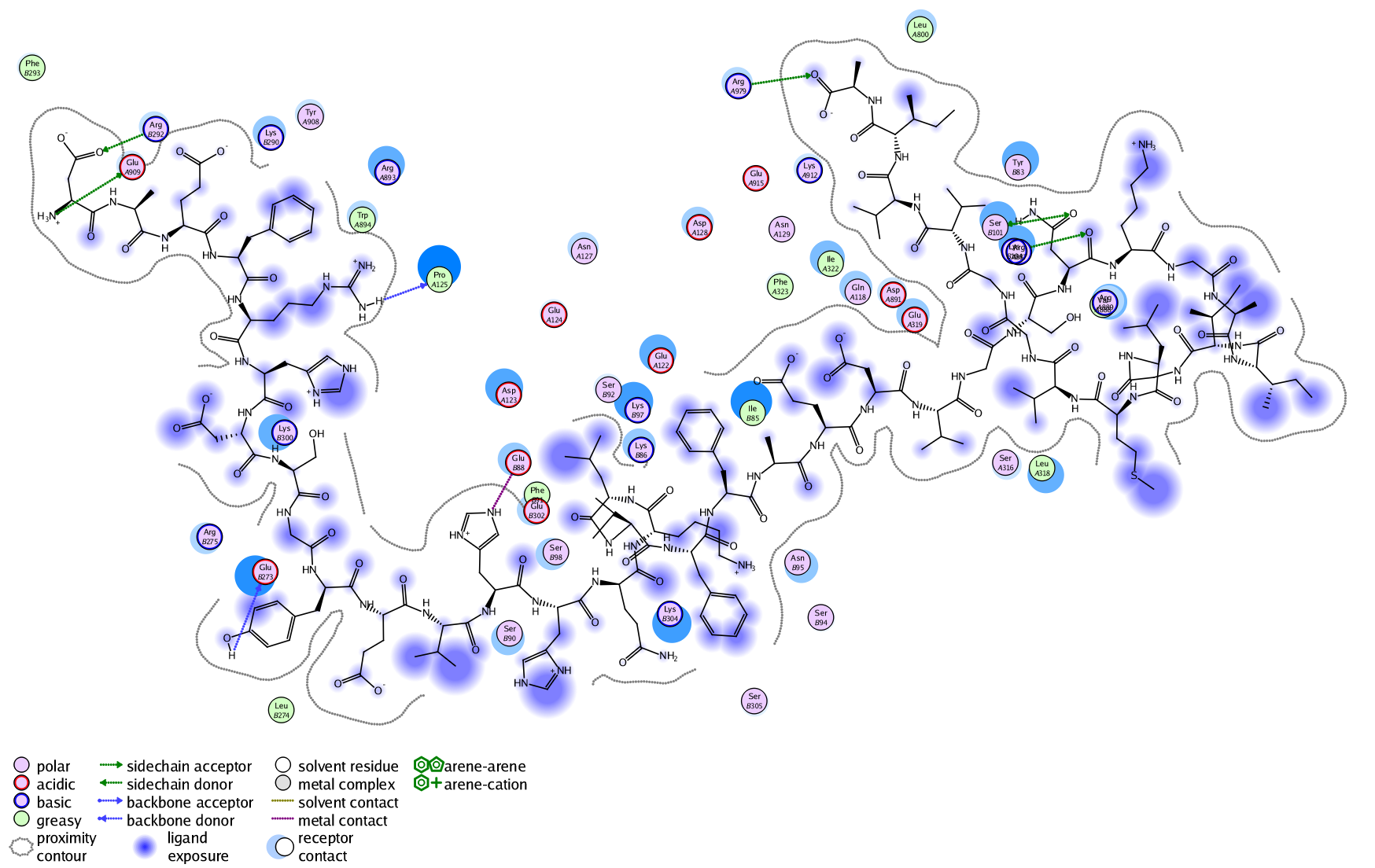


Figure S7. Intermolecular contacts of Aβ(1-42) and Na,K-ATPase for the model with the best score. Created by the molecular modelling software MOE.


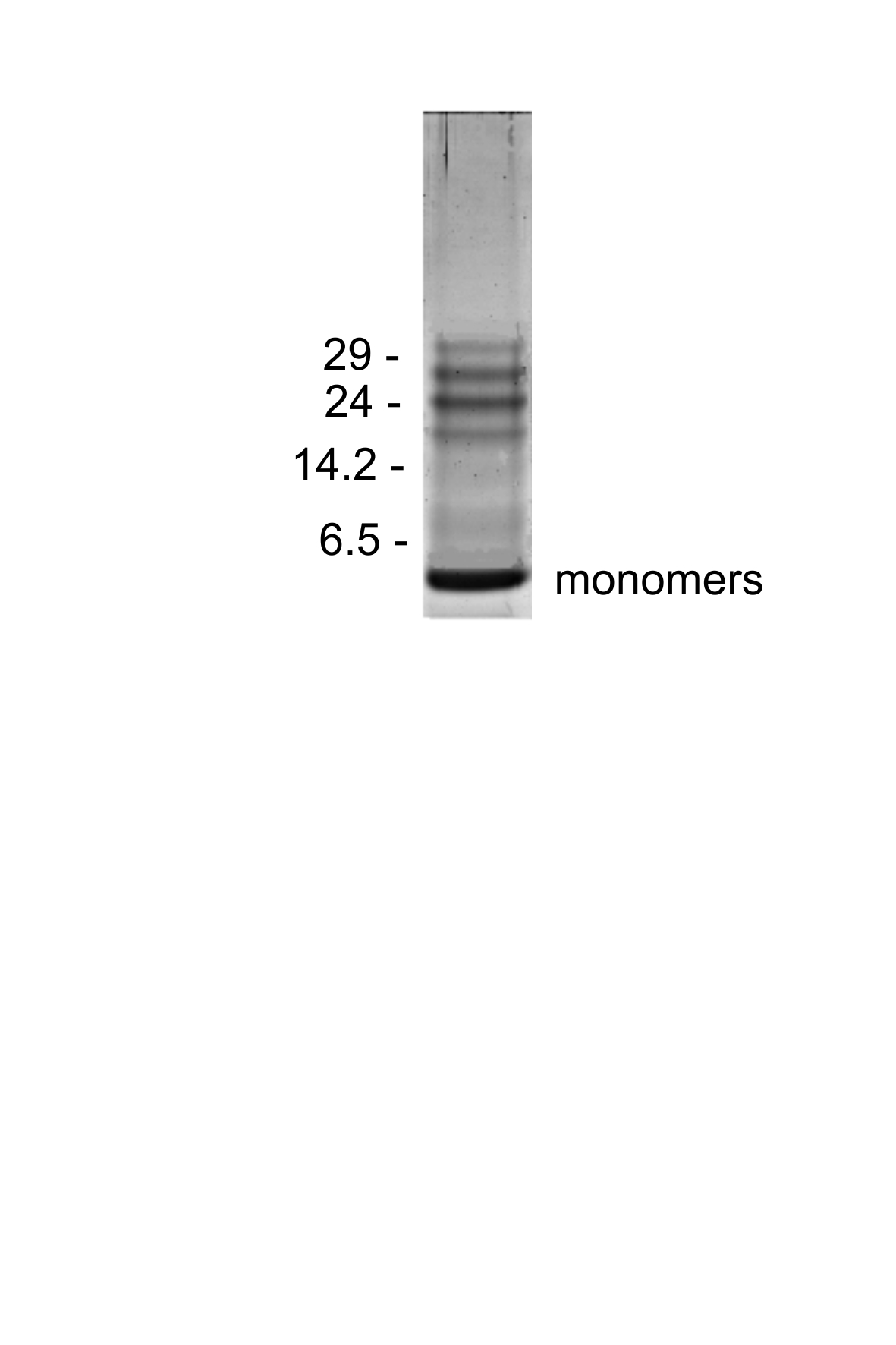


Figure S8. Coomassie-stained SDS-PAGE showing photo-cross-linked Aβ(1-42).

**1 2 3**

**kDa**

Figure S9. SDS-PAGE (**1,2**) and Western blot (**3**) characterization of Na,K-ATPase preparation isolated from duck salt glands. **1** molecular weight standards; **2** purified Na,K-ATPase; **3** visualization of Na,K-ATPase α1 subunit with anti-α1 antibodies.
